# Supplementary material for: Formulation of tunable size PLGA-PEG nanoparticles for drug delivery using microfluidic technology
Source: PLoS One. 2021 Jun 18;16(6):e0251821. doi: 10.1371/journal.pone.0251821 (PMC8213178; doi:10.1371/journal.pone.0251821)
Supplement: S1 File — (DOCX) [file pone.0251821.s001.docx]

**S1 File**

Formulation of tunable size PLGA-PEG nanoparticles for drug delivery using microfluidic technology

Adrianna Glinkowska Mares^1^, Gaia Pacassoni^1,2^, Josep Samitier Marti^1,3,4^, Silvia Pujals^1,3^, Lorenzo Albertazzi^1,5^

^1^Institute for Bioengineering of Catalonia (IBEC), The Barcelona Institute of Science and Technology (BIST), Carrer Baldiri Reixac 15-21, 08024 Barcelona, Spain.

^2^Department of Mechanical and Aerospace Engineering, Politecnico di Torino, Corso Duca degli Abruzzi, 10129 Torino, Italy

^3^Department of Electronic and Biomedical Engineering, Faculty of Physics, University of Barcelona, Carrer Martí i Franquès 1, 08028 Barcelona, Spain.

^4^Networking Biomedical Research Center in Bioengineering, Biomaterials and Nanomedicine (CIBER-BBN), 28029 Madrid, Spain

^5^Department of Biomedical Engineering, Institute for Complex Molecular Systems (ICMS), Eindhoven University of Technology, 5612AZ Eindhoven, The Netherlands

## Calculation of mixing time scale in the hydrodynamic flow focusing device

We estimated the mixing time $\tau_{mix}$ in our device to be 0.25 – 44 ms from the following equation^1^

$$\tau_{mix} \sim\frac{w_{f}^{2}}{4D} \approx\frac{w^{2}}{9D}\frac{1}{{(1+\frac{1}{R})}^{2}}$$

for D = 10^-9^ m^2^/s and w = 200 µm and used R flow ratios (0.0075 – 0.11).
