# Supplementary figures and images for: Formulation of tunable size PLGA-PEG nanoparticles for drug delivery using microfluidic technology
Source: PLoS One. 2021 Jun 18;16(6):e0251821. doi: 10.1371/journal.pone.0251821 (PMC8213178; doi:10.1371/journal.pone.0251821)

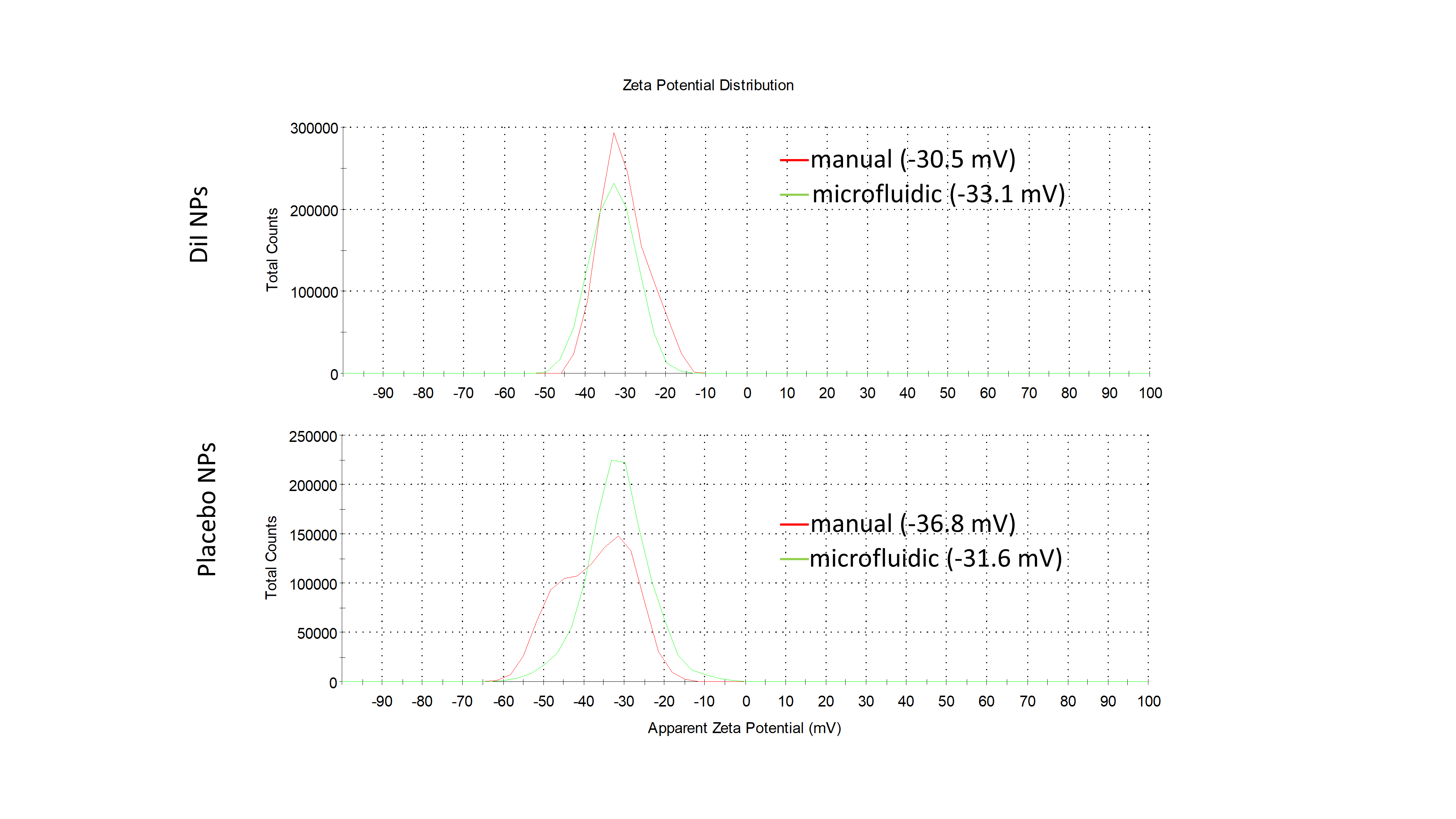

Supplement: S1 Fig — The average values (given in the legend) are between -36.8 mV to -30.5 mV what agrees with reported values for this polymeric compound. The graphs and mean values represent an average of triplicates. (TIF) [file pone.0251821.s002.tif]

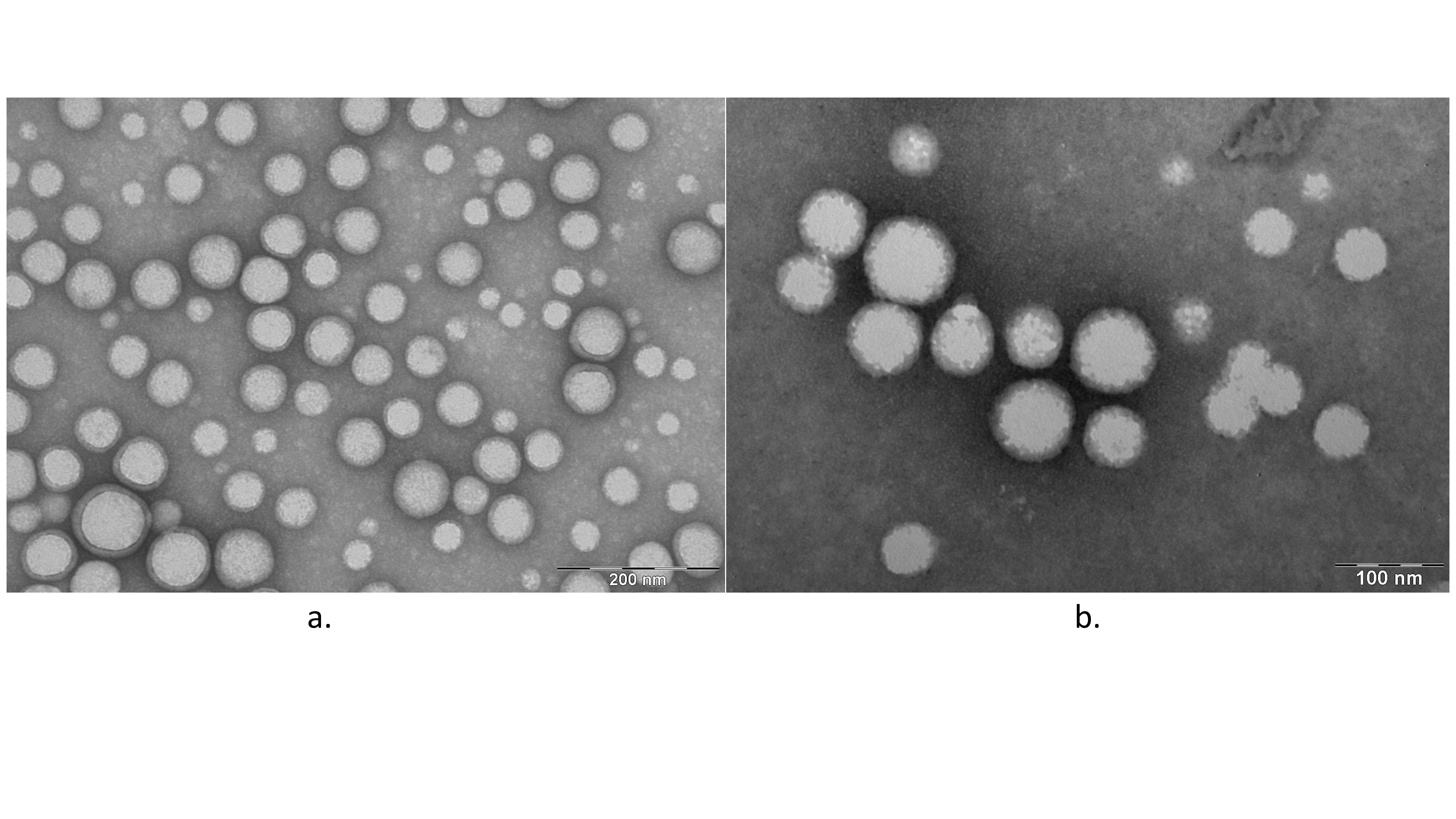

Supplement: S2 Fig — TEM image of NPs with distinctive core and corona, a. formulated with HFF @ S/AS 0.036, b. formulated by manual bulk mixing @ S/AS 0.1. (TIF) [file pone.0251821.s003.tif]

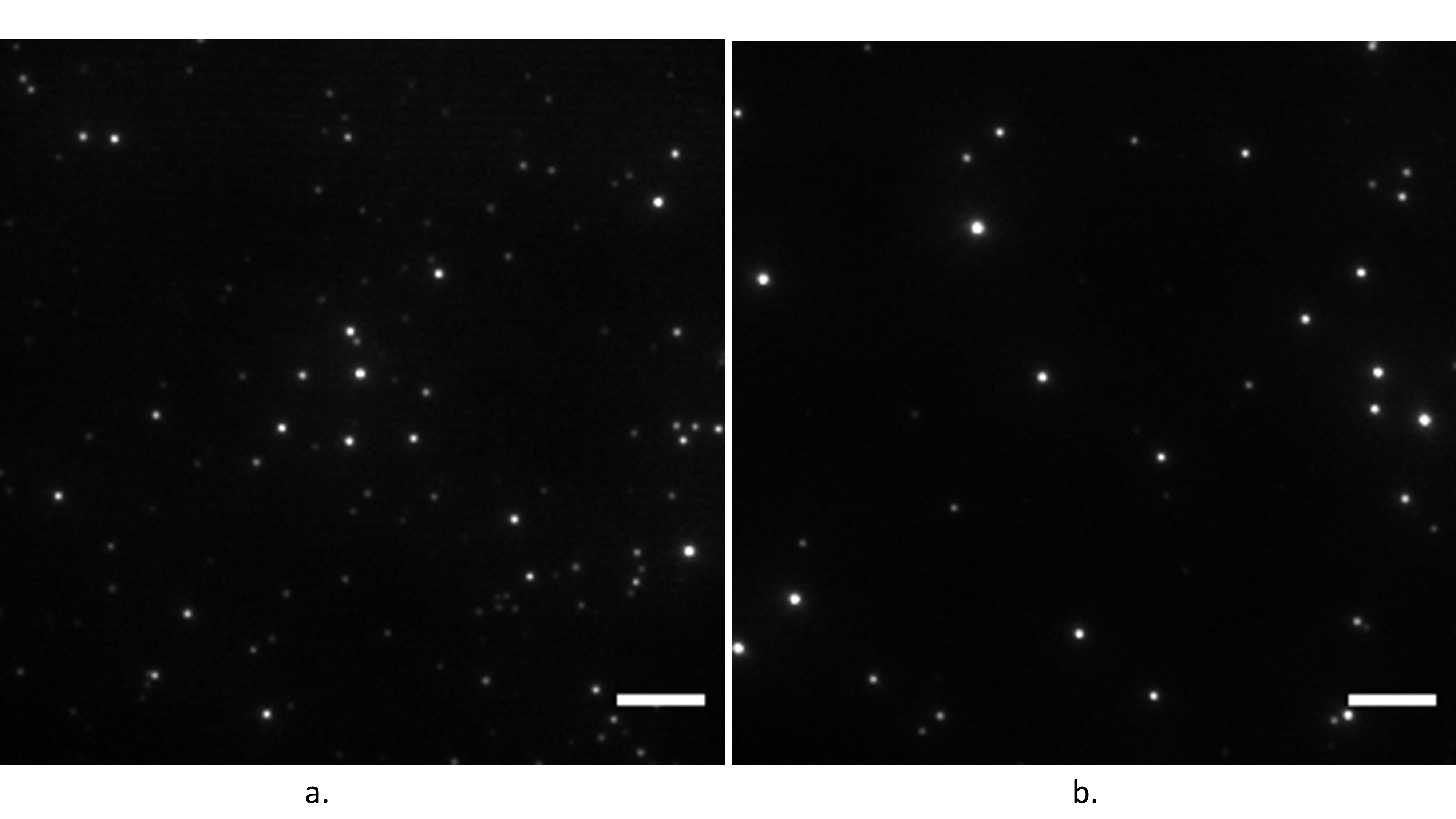

Supplement: S3 Fig — TIRF images of DiI loaded NPs formulated with HFF method, to compare fluorescence intensity between two formulations a. S/AS 0.095, particle diameter by DLS: 71m, b. S/AS 0.015, particle diameter by DLS 95 nm. Scale bar 5 μm. (TIF) [file pone.0251821.s004.tif]

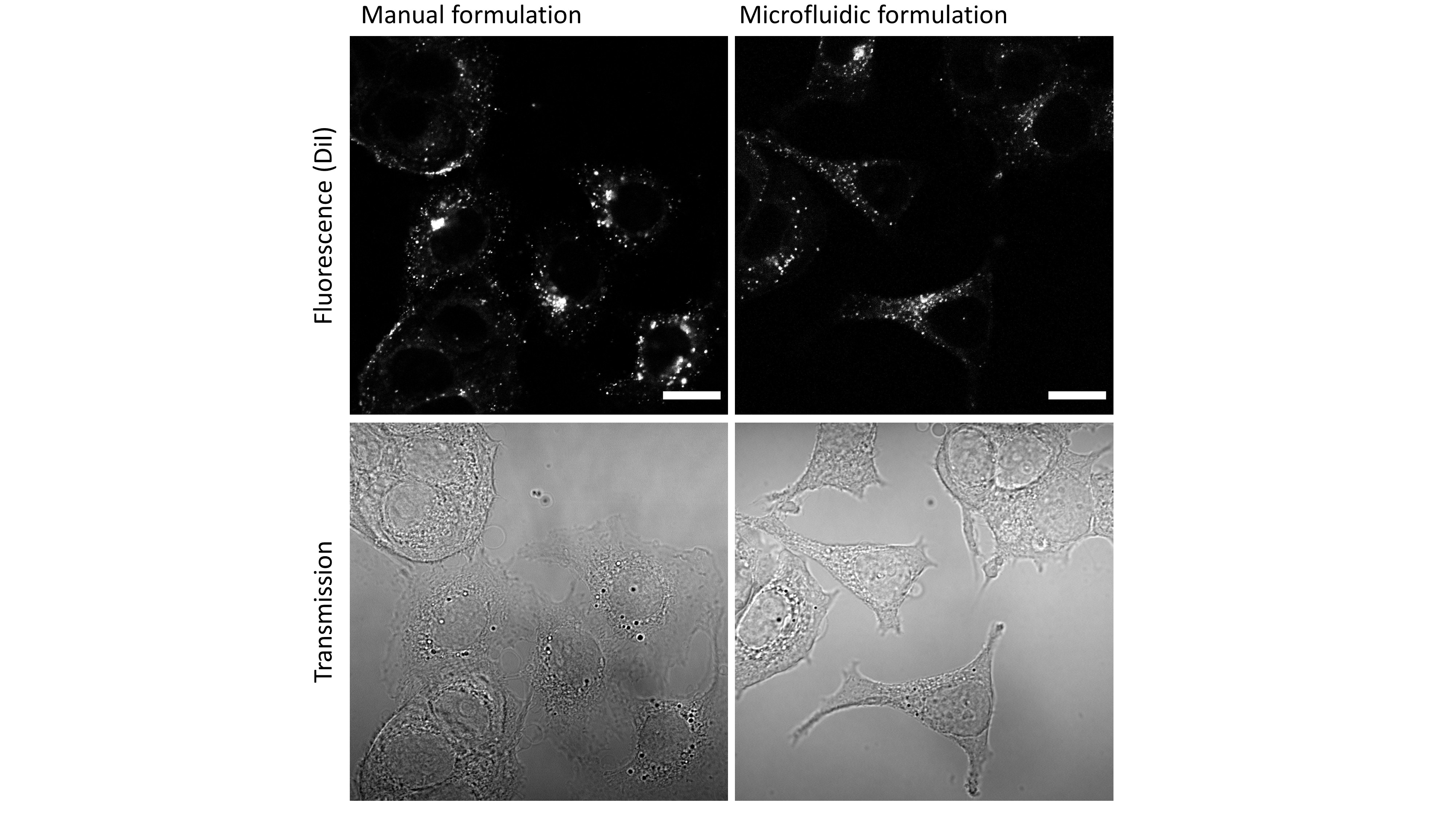

Supplement: S4 Fig — Top row: fluorescence images and bottom row: corresponding transmission images, scale bars 30 μm. (TIF) [file pone.0251821.s005.tif]

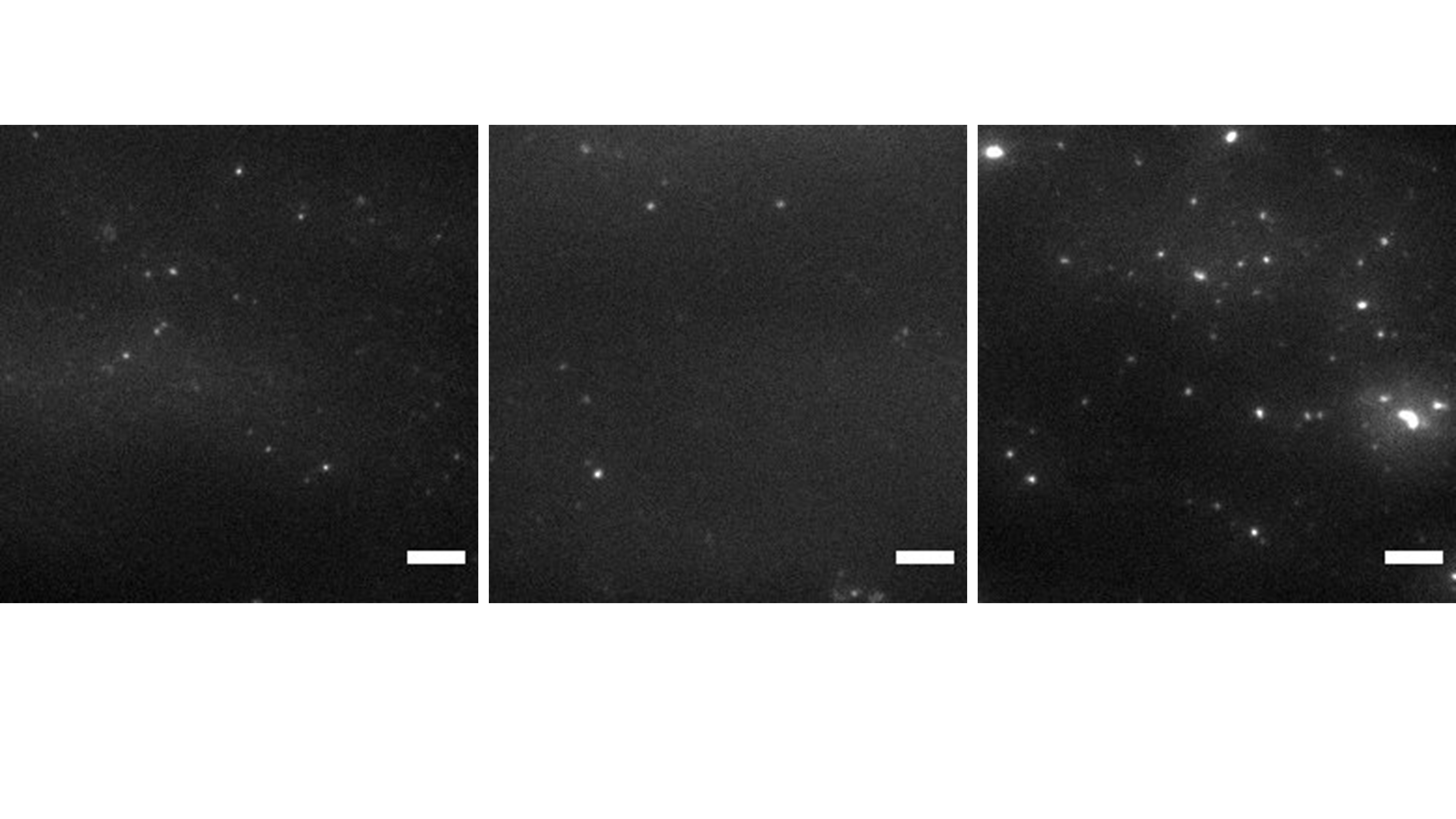

Supplement: S5 Fig — The particle fluorescence intensity is rather low due to the low EE, comparing for example to the S3 Fig. Scale bar 5 μm. (TIF) [file pone.0251821.s006.tif]

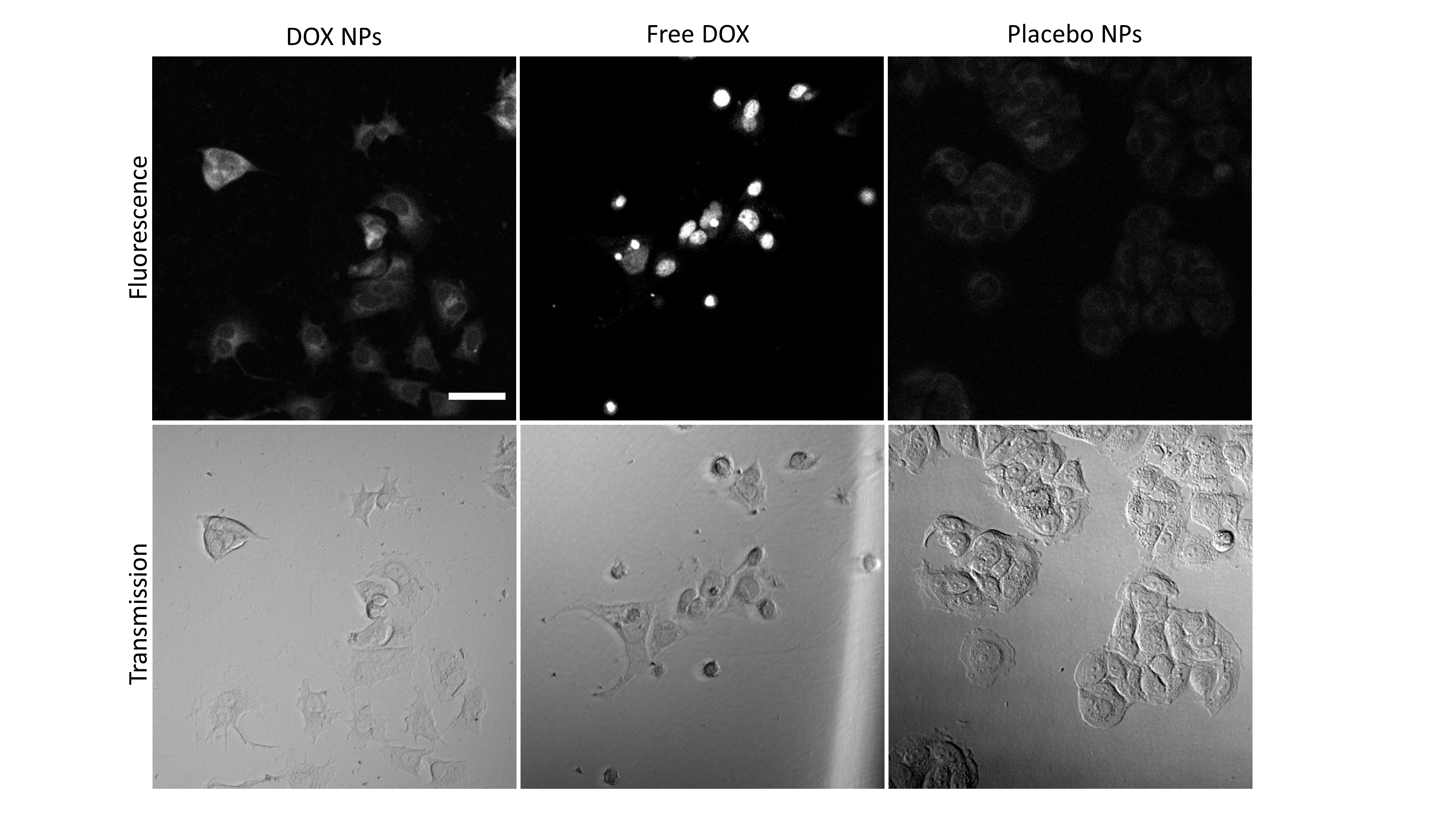

Supplement: S6 Fig — Fluorescence signal is detected in cell cytoplasm and around the nucleus for NPS loaded with DOX. On the other hand the free form of DOX is mostly concentrated in the nuclei, as can be seen in the central panel. PLGE-PEG NPs (right panel) show slight fluorescent signal in the excitation/emission corresponding to the DOX. Scale bar 100 μm. (TIF) [file pone.0251821.s007.tif]
