# Supplementary material for: Formulation of tunable size PLGA-PEG nanoparticles for drug delivery using microfluidic technology
Source: PLoS One. 2021 Jun 18;16(6):e0251821. doi: 10.1371/journal.pone.0251821 (PMC8213178; doi:10.1371/journal.pone.0251821)
Supplement: Attachment — Submitted filename: Rebuttal NPs_R2R.docx [file pone.0251821.s008.docx]

Dear Editor,

Please find attached the revised version of our manuscript. We want to thank the reviewers for their constructive criticism that helped to improve the article. We think we addressed all the issues raised and we hope that the manuscript is now suitable for publication.

Warmest regards

Lorenzo Albertazzi

**Reviewer #1: In this work, the authors described the preparation of PLGA-PEG nanoparticles by microfluidic technique. I appreciate the manuscript that focuses on the research close to a real application, not only for the very sophisticated things, however, cannot be repeated or large scale synthesis is rather doubtful. This is an example of the paper in which the guidelines of well-known PLGA-PEG NPs preparation is described, however, in the microfluidic channels. Nevertheless, I have found some drawbacks that still required corrections. Therefore, after a major correction and required corrections, the manuscript could be published in PLOS ONE.**

We thank the Reviewer for the kind words and interest in our manuscript, as well as for the instructive comments. We have now addressed all the comments and suggestions to increase the quality of the manuscript.

The author should discuss and compare their founding’s with these two papers already existing in the literature:
Journal of Colloid and Interface Science 475 (2016) 136–148
International Journal of Pharmaceutics 548 (2018) 530–539
Applied Nanoscience 8 (2018) 905–914

In our work we proposed a simple chip geometry, that as the Reviewer has noticed, is closer to an industrial translation than very sophisticated setups. For the same reason we worked on PLGA-PEG diblock copolymer, which is approved for parenteral administration.

The articles mentioned above by the Reviewer, discuss microfluidic formulation of NPs based on various polymers (PCL-b-PEO, (PEG-)PL-CL and (PEG-)PLGA) and obtained in diverse chip geometries. We have now included the suggested literature positions to the manuscript, confirming the reported size dependence for our PLGA-PEG NPs.

Here we would like to highlight that in our experiments we performed a thorough study of microfluidic and manual formulation for similar solvent and antisolvent ratios, as well as without use of auxiliary surfactants, which usually require additional purification steps, to match the EMA/FDA regulated dose limits (such as PVA).

Why authors choose the acetonitrile (ACN) for the preparation of NPs?

The choice of ACN is based on its compatibility with our chip (PDMS), polymer solubility and water miscibility. In the future we plan to explore other solvents (e.g. acetone, THF) in a borosilicate glass chip. We have explained now the choice of ACN in the materials and methods section.

I recommend comparing the mixing parameters of ACN with water and other solvents as it was shown in the paper: Colloids and Surf. B 2021, 201, 111598.
We added this reference to our manuscript, together with the following article:

Martina Pannuzzo, Bruno A. C. Horta, Carmelo La Rosa, and Paolo Decuzzi

*Macromolecules* **2020** *53* (10), 3643-3654

DOI: 10.1021/acs.macromol.0c00110

Now, we have added calculation of mixing time based on the solvent diffusivity in the system and our microchannel design parameters, specifying the values in the manuscript and the calculation in the SI.

Moreover, in future work, it is good to consider the more volatile solvent which is easier to remove from NPs, for instance, THF.

Yes, we definitely agree. As mentioned in the comment above, we were working on a borosilicate glass microfluidic chip that will allow such exploration.

DiI, this abbreviation is not described in the section about the encapsulation of fluorescent compounds.

We included now this information of materials and methods: 1,1'-Dioctadecyl-3,3,3',3'-Tetramethylindocarbocyanine Perchlorate (DiI, lipophilic cationic carbocyanine dye, Sigma Aldrich, 42364) into the indicated paragraph.

You should describe why there is so low DOX encapsulation?

I would recommend reading these two papers: Colloids Surfaces B Biointerfaces. 141 (2016) 187–195; Sci. Rep. 7 (2017) 1–12. I believe you can find the answer there.

Doxorubicin is a hydrophilic drug, and here we formulated NPS with PLGA hydrophobic core, able to encapsulate lipophilic molecules with higher efficiency than the hydrophilic ones. In the literature there are strategies to improve the DOX encapsulation into PLGA, by conjugation of DOX to hydrophobic molecules or by formulation based on emulsification. In our work we applied nanoprecipitation process and uniform parameters and procedures for the 3 encapsulated molecules (DOX, NR, DiI), revealing the encapsulation efficiency is based on the characteristics of these molecules.
Indeed, as expected, the DOX EE was the lowest, and modification of our process would be required to formulate NPs that could be considered for therapeutic application.

We added comment in the manuscript about this low EE values.

I won’t start the sentence with: Thanks to…

We have now rephrased such sentences

I am confused with the results of cellular uptake. Why authors didn’t show the uptake for NPs loaded with DOX?

Maybe, the reason for their low efficiency is correlated with the fact that these NPs cannot reach the nucleus of the cell?

Therefore, I will recommend repeating the preparation of NPs loaded with DOX to obtain the higher EE and, subsequently, their cellular uptake should be also conducted.

Our aim was to demonstrate that the selection of a drug to be encapsulated into PLGA-PEG NPs is limited by the molecule properties and it preferably should be chosen from poorly water-soluble drugs, which are a large group in the family of chemotherapeutics. Here we compared compounds that were fluorescent having DOX fulfilling this criterium.

For the illustration purpose we demonstrated the images of cells exposed to the DiI loaded NPs, as the DiI encapsulation was superior to other tested molecules. The low encapsulation of DOX resulted in poor imaging conditions, specifically in lower fluorescence intensity, than it was for the DiI. We believe that in the case of our experiments the cargo (DOX, NR, DiI) does not dictate the cellular uptake, but the nature of the NPs itself, which would be different if we were labelling independent polymer chains or worked with free drug.Therefor we only showed the DiI NPs in MCF-7 however, we reported the cell viability test including the DOX-loaded NPs. We have now added images of MCF-7 exposed to the DOX-loaded NPs to the SI.

As for the NPs reaching the nucleus: we believe that the administered NPs do not enter the nucleus but are located in the endosomes. NPs can release the drugs from there and the small molecules can easily cross the endosome and nuclear membrane reaching the target.

The relevant reviews in the field should be cited:
Adv Drug Deliv Rev. 2018 Mar 15;128:101-114, doi: 10.1016/j.addr.2017.12.015.
RSC Adv., 2019, 9, 2055-2072, DOI: 10.1039/C8RA08972H
Polym. Int. 68 (2019) 997-1014, [https://doi.org/10.1002/pi.5753](https://eur02.safelinks.protection.outlook.com/?url=https%3A%2F%2Fdoi.org%2F10.1002%2Fpi.5753&data=04%7C01%7C%7C064acf029e804a76de2f08d8ef774878%7Ccc7df24760ce4a0f9d75704cf60efc64%7C1%7C1%7C637522642886909091%7CUnknown%7CTWFpbGZsb3d8eyJWIjoiMC4wLjAwMDAiLCJQIjoiV2luMzIiLCJBTiI6Ik1haWwiLCJXVCI6Mn0%3D%7C1000&sdata=vDo8N8wWOK8JgtNc0kw8NNBUVjjCST7yiltk3X7u1Y8%3D&reserved=0)
Lab Chip, 2017, 17, 209-226, DOI: 10.1039/C6LC01049K
Nanoscale, 2016,8, 12430-12443, [https://doi.org/10.1039/C5NR07964K](https://eur02.safelinks.protection.outlook.com/?url=https%3A%2F%2Fdoi.org%2F10.1039%2FC5NR07964K&data=04%7C01%7C%7C064acf029e804a76de2f08d8ef774878%7Ccc7df24760ce4a0f9d75704cf60efc64%7C1%7C1%7C637522642886909091%7CUnknown%7CTWFpbGZsb3d8eyJWIjoiMC4wLjAwMDAiLCJQIjoiV2luMzIiLCJBTiI6Ik1haWwiLCJXVCI6Mn0%3D%7C1000&sdata=n9pmMdiHw4umBbUwdqM0maBZ3LELndRXjA4W6VAJwL4%3D&reserved=0)

We thank the Reviewer for the suggestions, now we have cited the mentioned articles.

**Reviewer #2: The synthesis of PLGA nanoparticles using microfluidics was compared to a bulk method of nanoparticle production. This has been done before for PLGA nanoparticles and so the authors should note the point of difference with their study and what has been done previously. A reference that is missing from this article that must be included is;
Streck S et al. (2019). Comparison of bulk and microfluidics for the formulation of functionalized poly-lactic-co-glycolic acid PLGA nanoparticles modified with cell-penetrating peptides of different architectures. International Journal of Pharmaceutics X** [**https://doi.org/10.1016/j.ijpx.2019.100030**](https://eur02.safelinks.protection.outlook.com/?url=https%3A%2F%2Fdoi.org%2F10.1016%2Fj.ijpx.2019.100030&data=04%7C01%7C%7C064acf029e804a76de2f08d8ef774878%7Ccc7df24760ce4a0f9d75704cf60efc64%7C1%7C1%7C637522642886919057%7CUnknown%7CTWFpbGZsb3d8eyJWIjoiMC4wLjAwMDAiLCJQIjoiV2luMzIiLCJBTiI6Ik1haWwiLCJXVCI6Mn0%3D%7C1000&sdata=dFsI%2B2n2D7d%2BlhNPqhCIe6LfVXQSzXaS%2BmvdDkTW%2BZg%3D&reserved=0)

We thank the Reviewer for the feedback on our manuscript, as well as for the instructive comments. We have now addressed all the comments and suggestions to increase the quality of the manuscript.

In our work we proposed a simple chip geometry that can be facile to scale out. We now have included the suggested reference as well as other references from the field, highlighting the differences of our work, which are: microfluidic formulation of PLGA-**PEG** nanoparticles, via surfactant-free nanoprecipitation, thoroughly compared at multipoint level to manual dropwise addition formulation and characterized by advanced microscopy techniques: TEM, TIRF. Additionally: evaluation of EE of 3 different fluorescent molecules with the focus on the comparison between the methods.

The authors encapsulated three different dye molecules and progressed to evaluate the toxicity of their formulation nanoparticles on a human breast epithelial cell line. This data was not mentioned in the abstract or conclusion however, and should be included.
We thank the Reviewer for this remark. We have added the bioevaluation to the abstract, introduction and conclusion parts.

How many independent batches were measured to obtain values for size and PDI of the nanoformulations?
We thank the Reviewer for rising this question. We have now reviewed the materials and methods section and specified that the results are based on measurements of **3** independent batches for each manual and microfluidic formulation and the results shown as a mean value with standard deviation

Why was zeta-potential not measured?

We thank the reviewer for pointing out the Z-P values. As the ZP for PLGA-PEG NPs is quite well described in the literature we have measured the ZP values only for selected formulations, and the results are now included into the SI.

The first paragraph of the introduction should be reduced because this information is VERY well established in the literature.

We have reduced the mentioned paragraph.

Specific comments

We thank for these comments, they are now addressed in our manuscript.

Abstract
Line 4. Review the use of English here “…allows to obtain…”.

Fig. 1. S and AS should be defined in the figure caption.

Fig. 2. HFF should be defined in the figure caption.

Paragraph “Particle diameter tunability using microfluidic chip” second sentence (“Faster diffusion yields smaller NPs…”). Please provide a reference citation or a citation to data in the manuscript for this statement.

“It is especially important to control the particle size, as their performance and cellular uptake depends on this feature.” Please provide a reference citation for this statement.

“This is a common strategy to encapsulate drugs into PLGA formulations.” Please provide reference citations for this statement.

Materials and methods, page 1, line 7 and elsewhere. Please correct ‘miliQ water’ to ‘purified water’.

**Reviewer #3: The authors described the comparison of the PLGA-based nanoparticles prepared using the typical manual bulk mixing and a microfluidic chip-assisted nanoprecipitation. Peglated PLGA nanoparticles prepared by both methods have been widely documented in the literature. The authors claimed the tuneability of the particle size by microfluidic method. Again this is already very well-documented in the literature, particularly for PLGA nanoparticles. Therefore, it is difficult to extract the novelty of this study. It maybe beneficial for the authors to re-consider the focus of the study. Specific comments are shown below:**

We thank the Reviewer for the feedback on our manuscript, as well as for the instructive comments. We have now addressed all the comments and suggestions to increase the quality of the manuscript.

We have included some references to highlight the novelty of our manuscript in comparison to the reported literature, which are:

1) microfluidic formulation of PLGA-**PEG** nanoparticles, via surfactant-free nanoprecipitation, thoroughly compared at multipoint level to manual dropwise addition formulation
2) the single particle characterizion by advanced microscopy techniques: TEM, TIRF allowing to discuss intra-batch heterogeneity in both formulation types
3) Additionally the evaluation of EE of 3 different fluorescent molecules with the focus on the comparison between the methods.

The first section of the Results and discussion, namely “Microfluidic hydrodynamic flow focusing and manual bulk formulation”, does not seem to be based on any data. It is not clear what the purpose of this section.

We thank the Reviewer for this comment. This section aims to introduce information about the microfluidic chip that we used, as well as to illustrate the two formulation methods and impact of antisolvent flow rate changes on the solvent concentration, based on the data retrieved from the simulation. We have changed the name of this section to match better its content

In this section, the authors claimed that CFD simulation was used to generate the Figure 1c. However, there was no CFD described in the method section. No real data to confirm the CFD prediction/simulation.

We thank the Reviewer for this comment. The submitted manuscript contains CFD simulation description in the materials and methods section.

The following information was provided:

*Comsol Multiphysics simulation*

Comsol Multiphysics 5.3 software was used for the CFD simulations. The chip mesh was generated from the AutoCAD design (the same as used for the acetate mask). The simulation was performed for fluid laminar flow in stationary conditions to observe the transport of diluted species upon different flow rates. Water and Acetonitrile properties at RT and 1 atm were introduced to perform the computation. The parameters used for the computation were as following: dynamic viscosity of ACN: 0.389 mPas, the density of ACN: 0.786 g/mL, the properties of water were selected automatically from the software library.

The presented output of the CFD simulation serves an illustrative purpose, as we did not measure the stream width changes upon the actual formulations. However, we observed the expected response of the solvent stream width to the changing flow rate of the antisolvent stream in other, unrelated experiments.

In section named “Particle diameter tunability using microfluidic chip” the authors only used one S/AS ratio for the conventional bulk method, which achieved similar particle size as the microfluidic method, but no other S/AS was used in the bulk method. This limited the quality of the comparison considering the significant differences in throughput rate of the two method. Such limited comparison seem inadequate for drawing any conclusion.

We thank the Reviewer for this comment; however, we believe that it is a result of a misunderstanding. The figure 2 represents Six points resulting from manual bulk formulations at various S/AS performed in triplicate each and corresponding to the microfluidic formulations.

The cellular studies were only performed on microfluidic particles, but not particles prepared by bulk method.

We thank the Reviewer for this remark. Indeed, we focused mostly on the tests of microfluidic formulation NPs, as this method is still less common and reported and represent the focus of the paper, while the manual formulation is well assessed and demonstrated to be biocompatible. We have now added images of cells exposed to manually formulated NPs into the SI.

The EE and single particle analysis also indicated that there is no significant difference in bulk and microfluidic method when the same A/SA ratios were used. These findings that are directly relevant to the comparison are not mentioned in the conclusion.

We thank for this comment. We now have added this information to the conclusions.

No statistical analysis was described in the method section.

We thank the Reviewer for rising this question. We have now reviewed the materials and methods section and specified that the results are based on measurements of **3** independent batches for each manual and microfluidic formulation and the results shown as a mean value with standard deviation
